# Characteristics of Ground Reaction Force Variability During Walking in Post-Stroke Patients

**DOI:** 10.3390/s25226940

**Published:** 2025-11-13

**Authors:** Daiki Naito, Yusuke Sekiguchi, Keita Honda, Midori Miyagi, Takeshi Yamaguchi, Toshiaki Nishi, Hide Matsumoto, Yuzuki Nakai, Yoshihiro Sasaki, Jun-Ichi Hayasaka, Daiki Haruyama, Koji Watanabe, Satoru Ebihara

**Affiliations:** 1 Department of Rehabilitation Medicine, Tohoku University Graduate School of Medicine, 1-1 Seiryo-machi, Aoba-ku, Sendai 980-8574, Miyagi, Japan; keita.honda.t2@tohoku.ac.jp (K.H.); midori.miyagi.b5@tohoku.ac.jp (M.M.); satoru.ebihara.c4@tohoku.ac.jp (S.E.); 2 Department of Rehabilitation, Southern Tohoku Second Hospital, 6-95 Yatsuyamada, Koriyama 963-8052, Fukushima, Japan; mf2402@isu.ac.jp (D.H.); koji.watanabe@mt.strins.or.jp (K.W.); 3 Research Management Center, Organization for Research Promotion, Tohoku University, 2-1-1 Katahira, Aoba-ku, Sendai 980-8577, Miyagi, Japan; yusuke.sekiguchi.b2@tohoku.ac.jp; 4 Graduate School of Dentistry, Tohoku University, 4-1 Seiryo-machi, Aoba-ku, Sendai 980-8575, Miyagi, Japan; 5 Department of Rehabilitation, Kumamoto Health Science University, 325 Izumi-machi, Kita-ku, Kumamoto 861-5533, Kumamoto, Japan; 6 Department of Finemechanics, Graduate School of Engineering, Tohoku University, Sendai 980-8579, Miyagi, Japan; takeshi.yamaguchi.c8@tohoku.ac.jp (T.Y.); toshiaki.nishi.b3@tohoku.ac.jp (T.N.); hide.matsumoto.r4@dc.tohoku.ac.jp (H.M.); nakai.yuzuki.r8@dc.tohoku.ac.jp (Y.N.); 7 Research Institute for Electromagnetic Materials, Tomiya 981-3341, Miyagi, Japan; sasaki@denjiken.ne.jp (Y.S.); hayasakaj@denjiken.ne.jp (J.-I.H.)

**Keywords:** stroke, gait, ground reaction force, variability

## Abstract

**Highlights:**

**What are the main findings?**

- Paretic-side anterior–posterior ground reaction force variability during pre-swing phase was lower than in age-matched control, independent of speed.
- The reduction in GRF variability during walking was suggested for maintaining gait and avoiding falls in post-stroke patients.

**What is the implication of the main finding?**

- GRF variability may reflect the adaptive gait control of participants.
- Rehabilitation that permits GRF variability may be beneficial for post-stroke patients with mild deficits.

**Abstract:**

Gait impairment in post-stroke patients increases the risk of falls, but the role of ground reaction force variability (GRF variability) in controlling gait stability remains unclear. The objectives of this study were (1) to clarify the differences in GRF variability during walking between post-stroke patients and age-matched controls and (2) to identify the differences in GRF variability between post-stroke patient fallers and non-fallers. Sixteen post-stroke patients (age: 72.19 ± 8.54, six female, four fallers: age: 71.75 ± 11.32, twelve non-fallers: age: 72.33 ± 8.03) and nineteen age-matched controls (age: 68.63 ± 5.73, nine female) participated. GRF variability was measured using shoe sensors during walking. After adjusting for walking speed, the anterior–posterior (AP) GRF variability on the paretic side in the 91–100% stance phase was significantly lower in the post-stroke patients (*F* = 3.721, *p* = 0.038). This phase’s AP GRF variability was not correlated with Berg Balance Scale scores. Furthermore, the faller group in stroke patients showed the AP GRF variability on the paretic side was lower in the 41–50% (*W* = 17, *p* = 0.045), 51–60% (*W* = 16, *p* = 0.045), 61–70% (*W* = 16, *p* = 0.045), and 91–100% (*W* = 23, *p* = 0.045) sub-stance phases. After adjusting for sex and orthosis, the sensitivity analysis showed no significant intergroup difference. This suggested an adaptive control mechanism for maintaining gait and avoiding falls in post-stroke patients.

## 1. Introduction

Strokes often result in motor paralysis, balance deficits, and cognitive dysfunction [1]. These impairments lead to gait deviations such as reduced walking speed, decreased cadence, shorter step length, and decreased toe clearance on the affected side [2,3,4,5]. These gait deviations contribute to an increased risk of falling [6]. The fall incidence among post-stroke patients is high, reaching approximately twice that of age-matched healthy adults [7]. Falls in this population often result in injury and fracture, leading to reduced mobility [8,9,10]. Furthermore, while the one-year mortality rate following a proximal femoral fracture is 10.8% for the general population, the rate for post-stroke patients with a femoral fracture is significantly higher, reaching 24.8% [11]. Crucially, falls most frequently occur during walking; therefore, improving gait stability is a critical challenge in stroke rehabilitation.

Stable gait requires effective stance–limb control in response to environmental changes and sudden perturbations. Healthy individuals maintain this stability by increasing the magnitude of the ground reaction force (GRF) and modifying the GRF vector angle in response to walking perturbations [12,13]. The vertical (V) GRF plays a role in supporting body weight during the loading response and decelerating the body’s center of mass during the latter half of the stance phase while walking [14]. A positive correlation has been reported between the V GRF of the paretic and non-paretic limbs during static standing and the Berg Balance Scale (BBS) score in post-stroke patients [15]. Furthermore, older adults with a history of falls had a longer time to reach the first V GRF peak compared to older adults without a history of falls [16]. These reports suggest that GRF could serve as an indicator of gait stability. Previous studies have reported reduced V and anterior–posterior (AP) GRF on the paretic side of post-stroke patients compared to healthy individuals [17,18]. V GRF and AP GRF have been shown to reduce, particularly during the latter half of the stance phase [19,20,21,22]. The anterior GRF during the latter half of the stance phase indicates propulsion force and is an important factor in walking speed [23]. The latter half of the stance phase requires complex lower-extremity control as it simultaneously involves supporting limb propulsion and contralateral limb initial contact [24,25,26]. Thus, given that GRF is reduced during the latter half of the stance phase in post-stroke patients, it is presumed that they have difficulty maintaining stability during terminal stance, which requires complex control. A critical consideration is that the observed differences in GRF between post-stroke patients and healthy individuals are often significantly influenced by reduced walking speed, which is a key clinical manifestation of strokes [23]. Consequently, determining whether GRF differences are significantly a direct pathological consequence or epiphenomenon of reduced walking speed is a persistent methodological and clinical challenge in stroke gait analysis.

Maintaining movement stability requires adaptive modifications, necessitating a certain range of movement variability, which is influenced by aging, pathology, and neurological factors [27,28]. Among post-stroke patients, increased variability in trunk acceleration and stride time during walking has been linked to a history of falls, demonstrating its utility as an assessment for fall risk [29,30,31]. In contrast, GRF variability reflects the consistency and adaptability of gait control rather than its magnitude. Increased variability may indicate greater flexibility and adaptability under challenging conditions, such as walking on unstable surfaces [32], whereas excessive variability in steady walking environments may reflect impaired motor control. Therefore, analyzing GRF variability provides complementary information about gait stability and motor control that cannot be captured by conventional mean or peak GRF parameters. Nevertheless, the relationship between GRF variability and falls remains unclear. Furthermore, while post-stroke patients exhibit greater variability in maximum AP GRF during the latter half of the stance phase compared to healthy controls [33], this prior investigation was limited to the variability of a single point (maximum AP GRF). A study that compared the entire support phase in elderly individuals and young, healthy adults—time-normalizing the data and examining whether variance differed across segments—demonstrated phase-specific differences in GRF variability [34]. These results suggest the need to compare changes in GRF variability throughout the entire gait cycle. A comparison of GRF changes across stance phases revealed that post-stroke patients have exhibited an earlier onset of the AP GRF peak compared to healthy individuals [35]. Furthermore, GRF variability is influenced by walking speed [36]. Adjusting for walking speed may reveal differences in GRF variability between stroke survivors and healthy individuals. Thus, GRF variability in post-stroke patients may exhibit different patterns than those of healthy individuals during specific stance phases. To our knowledge, no prior study has examined the characteristics of GRF variability in the stance sub-phase in post-stroke patients or its relationship with balance ability. We hypothesized that post-stroke patients (especially faller) increase AP GRF variability during the terminal stance to pre-swing phase, a phase that requires complex propulsive control to maintain gait control.

The objectives of this study were (1) to clarify the characteristics of GRF variability in the stance sub-phase in post-stroke patients by comparing their AP and V GRF variability during walking with that of age-matched controls, (2) to investigate the relationship between GRF variability in stance sub-phase and relevant clinical balance assessments, and (3) to identify the characteristics of GRF variability of stance sub-phase in the faller and non-faller groups of post-stroke patients.

This study is novel in that it utilizes a wearable sensor shoe system to assess GRF variability during walking in a near-real-world environment. Unlike conventional laboratory-based measurements using force plates, this system allows for continuous assessment of both vertical and anterior–posterior GRFs during natural gait. This methodological approach provides a more ecologically valid understanding of how post-stroke individuals adapt their gait to maintain stability in daily life.

This approach may help us understand how post-stroke patients maintain stable walking. This paper is organized as follows. Section 2 describes the methods in this study. Section 3 presents the results. Section 4 discusses the findings and their implications. Finally, Section 5 concludes the paper.

## 2. Materials and Methods

### 2.1. Participants

A total of 20 post-stroke patients (13 males, age range of 55–88 years) admitted to our convalescent rehabilitation ward and 20 age-matched controls (10 males, age range of 60–77) were included this study. The inclusion criteria for the post-stroke patients were as follows: (1) hemorrhage or infarction in the supratentorial region on unilateral side by computed tomography (CT) or magnetic resonance imaging (MRI) and diagnosis of cerebral hemorrhage of infraction, (2) patients aged 20 years old or older, and (3) patients who were able to walk in a straight line for 30 consecutive gait cycles at their self-selected optimal speed without the assistance of another person and the use of lower limb orthoses. The inclusion criteria for age-matched controls also included (2) and (3). The exclusion criteria for post-stroke group were as follows: (1) comorbid musculoskeletal disorders that impede walking performance; (2) difficulty in communicating and understanding instructions because of higher brain dysfunction, cognitive decline, or aphasia, which makes executing the study challenging; (3) history of neurological disease. The exclusion criteria for age-matched control group were as follows: (1)–(4) history of falls within the past year. Participants provided written informed consent.

### 2.2. Clinical Assessment

In post-stroke patients, motor function was assessed using Fugl–Meyer Assessment of lower extremity (FMA-L) [37] and Stroke Impairment Assessment Set (SIAS) to determine scores for lower extremity motor function (hip flexion test, knee extension test, and foot tap test) [38]. Balance ability was assessed using Berg Balance Scale (BBS) [39]. Short Falls Efficacy Scale-International (Short FES-I) was used as measurement of fear of falling [40]. This scale has been shown to be associated with the occurrence of falls [41]. Fall status was confirmed by telephone for every 1 month over 6 months in post-stroke patients, and post-stroke patients were assigned in faller group and non-faller group. We defined fall event as “an undesired contact of any body part (other than the feet) with the ground or a lower surface” [42].

### 2.3. Gait Assessment

**Figure 1:**
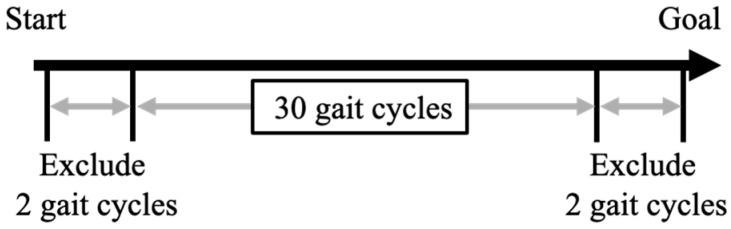
Gait task.

The motor task required participants to walk in a straight-line for 34 gait cycles at their self-selected comfortable speed. Participants completed one trial of the walking measurement. The continuous 30 gait cycles were subsequently used for analysis, excluding the initial and final two gait cycles immediately after the start and before the end of walking, respectively [43] (Figure 1). This method was deemed valid for measuring GRF variability during gait, as previous research has indicated that recording 20 or more gait cycles enables the measurement of kinematic parameter variability during walking with high intra-rater reliability [44].

### 2.4. Shoe Sensor System

**Figure 2:**
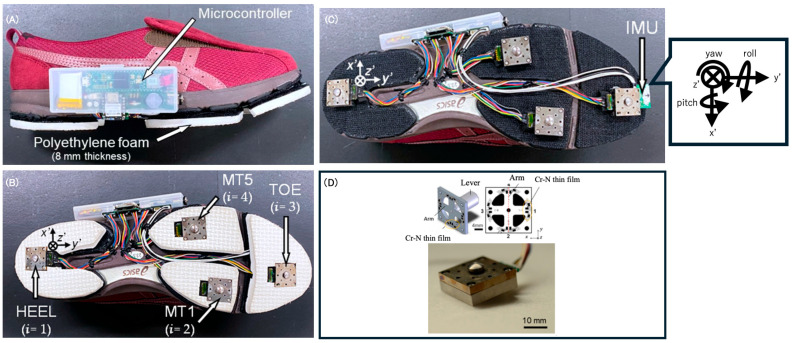
The shoe sensor used for gait measurement. (**A**) The external appearance of the sensor shoe. (**B**) The location of the ground reaction force (GRF) sensors embedded in the sole. HEE: The heel, MT1: the first metatarsal bone, MT5: the fifth metatarsal bone, TOE: the toes. (**C**) An illustration of the inertial sensors integrated into the sole. IMU: Inertial measurement unit. (**D**) Internal structure of the sensor of a single sensor unit.

We assessed GRF during straight walking using the shoe sensor system [45,46] (Figure 2). This shoe sensor system has four triaxial force sensors (at the heel, first and fifth metatarsal heads, and toe) able to measure GRF outside of a laboratory setting [45,46]. The force sensor is composed of a stainless-steel housing and a force-sensing lever (Research Institute for Electromagnetic Materials, Miyagi, Japan). GRFs were measured along the anterior–posterior (AP), vertical (V), and medial–lateral axes. GRF data from the paretic and non-paretic side of the post-stroke patients were used in the analysis. For the control group, GRF data from the non-dominant leg (all left side) was analyzed. Data from the non-dominant leg was used because it has been shown to exhibit smaller changes in GRF in response to variations in walking speed [47]. Given our aim to investigate excluding the influence of walking speed, this study utilized data from the non-dominant limb. Furthermore, to eliminate the influence of shoe differences on gait mechanics, bilateral sensor shoes were used for the control group during measurement. The sampling frequency was 400 Hz. The maximum measurement range of the force sensor was ±500 N for the AP and medial–lateral axes and ±1000 N for the V axis. The ICC for V GRF during the 1–10% sub-stance phase was 0.49, indicating low reliability. This is likely due to the influence of foot contact points during heel strike. Therefore, the V GRF data for this segment was interpreted with caution.

The inertial measurement unit (IMU) (9-DOF Absolute Orientation IMU Fusion Breakout-BNO055; Aafruit, NY, USA) with a sampling frequency of 70 Hz was mounted at the toe of the shoe. It was housed within polyethylene foam embedded in the sole of the shoe for protection from external impact. Both the force sensor and the IMU were connected to a microcontroller (Teensy 3.6, SparkFun, Electronics^®^, Niwot, CO, USA). The microcontroller was connected to a trigger that was activated by pressing a switch. The system allowed for the measurement data to be recorded onto a secure digital card.

### 2.5. Data Processing

Data analysis in this study was performed using MATLAB version R2023b (the MathWorks, Inc., Natick, MA, USA, https://www.mathworks.com (accessed on 12 February 2024)). Data processing followed the methods of Yamaguchi et al. and Matsumoto et al., who used the same shoe sensor [45,46]. For calibration, the shoe sensor was connected to a personal computer and placed on a level surface prior to participant testing. Acceleration calibration was performed using the Newton iterative optimization algorithm [46]. This aligned the resultant vector of the three-axis acceleration data (measured by the shoe sensor’s IMU in a static state) with the acceleration due to gravity (9.8 m/s^2^). The Newton iterative optimization algorithm is an iterative method that calculates the solution that minimizes the calibration coefficients and bias values. This minimizes the error between the resultant of the three-axis acceleration data and the acceleration due to gravity (9.8 m/s^2^). Using this algorithm, the sensor shoes were calibrated when placed on a level surface without a participant so that the resultant three-axis acceleration equaled the acceleration due to gravity (9.8 m/s^2^). The corrected acceleration values for each axis ($\alpha_{x0},\alpha_{y0},\alpha_{z0}$) were thus determined. Next, the initial angles of the IMU $\theta_{0}$ around the $x^{\prime }$-axis and $\varphi_{0}$ around the $y^{\prime }$-axis at the initial static position of the IMU sensor were calculated using calibrated acceleration data ($\alpha_{x0},\;\alpha_{y0},\;\alpha_{z0}$) according to the following equations:

(1) $$\theta_{0}=-arctan\left(\frac{\alpha_{z0}}{\alpha_{x0}}\right)$$

(2) $$\varphi_{0}=arctan(\frac{\alpha_{x0}}{\sqrt{\alpha_{y0^{2}}+\alpha_{z0^{2}}}})$$

The initial angle around the $z^{\prime }$-axis ($\psi_{0}$) was set to 0°.

**Figure 3:**
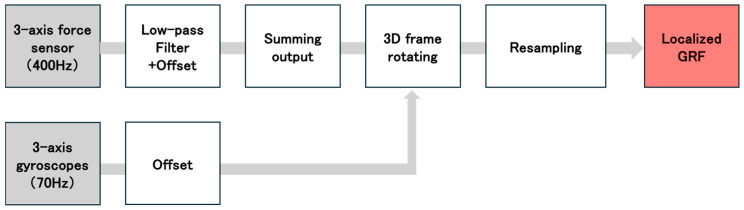
The algorithm for ground reaction force (GRF) data analysis. A flowchart illustrating the GRF data analysis process. The GRF data were first corrected using the angular velocity data measured by the inertial sensors, and then, the final values were calculated.

Figure 3 shows the analytical algorithm used to calculate the GRF obtained from the individual sensors at each location [45,46]. We analyzed the medial–lateral GRF ($F_{X^{\prime }i}$), the AP GRF ($F_{y^{\prime }i}$), and V GRF ($F_{z^{\prime }i}$), which was recorded during the stance phase. The subscript i denotes the location of each sensor on the foot sole: 1 = heel, 2 = first metatarsal head, 3 = fifth metatarsal head, and 4 = toe. To remove noise from the data, a fourth-order Butterworth low-pass filter with a cutoff frequency of 50 Hz was applied. Next, the GRF data from each sensor location ($F_{x^{\prime }i},\;F_{y^{\prime }i},\;F_{z^{\prime }i}$) were transformed into the global horizontal and vertical coordinate system ($F_{Xi},\;F_{Yi},\;F_{zi}$) using the following equations [46]:

(3) $$\left[\begin{array}{c} F_{xi}\\ F_{yi}\\ F_{zi} \end{array}\right]=\left[\begin{array}{c} \begin{array}{ccc} \cos\varphi & 0 & \sin\varphi \\ 0 & 1 & 0\\ -\sin\varphi & 0 & \cos\varphi \end{array} \end{array}\right]\left[\begin{array}{ccc} 1 & 0 & 0\\ 0 & \cos\theta & -\;\sin\theta \\ 0 & \sin\theta & \cos\theta \end{array}\right]\left[\begin{array}{c} F_{x^{\prime }i}\\ F_{y^{\prime }i}\\ F_{z^{\prime }i} \end{array}\right]$$

The local GRF at each sensor location ($F_{Xi},F_{Yi},F_{zi}$) were transformed into the global horizontal and vertical coordinate system. The GRF for entire sensor shoe (Fx, ℱy,Fz) was calculated by summing the forces from all sensor locations using the following equation [46]:

(4) $$\left[\begin{array}{c} F_{x}\\ F_{y}\\ F_{z} \end{array}\right]=\sum_{i\;=\;1}^{i\;=\;4}\left[\begin{array}{c} F_{xi}\\ F_{yi}\\ F_{zi} \end{array}\right]$$

**Figure 4:**
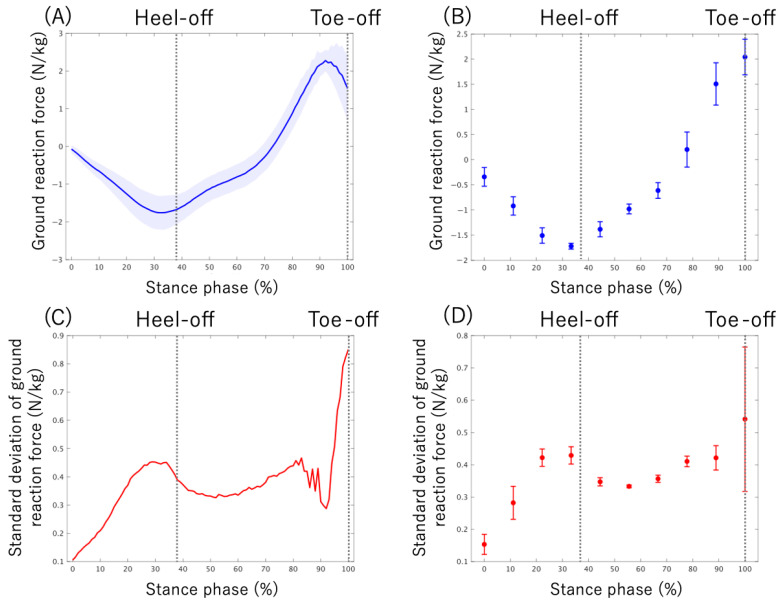
Changes in the ground reaction force (GRF) during the stance phase, shown as a representative example of a healthy individual. The dashed lines indicated the stance phase events (heel-off, toe-off). (**A**) The time course of the GRF normalized by body weight (*y*-axis) over the stance phase (*x*-axis). The shaded area indicates the standard deviation of the GRF. (**B**) The GRF magnitude averaged for every 10% of the stance phase. The error bars indicate the standard deviation of the GRF for each phase. (**C**) The time course of GRF variability (*y*-axis) over the stance phase (*x*-axis). The standard deviation of the GRF was taken as the value of GRF variability. (**D**) GRF variability averaged for every 10% of the stance phase. The error bars indicate the standard deviation of GRF variability.

The stance phase of walking was identified using V GRF ($F_{z}$) from each sensor. Stance phase was defined as the moment when the V GRF ($F_{z}$) exceeded 15 N, and stance termination was defined as the moment when the V GRF ($F_{z}$) dropped below 15 N [45]. Previous studies targeting stroke survivors have defined the stance phase using a force threshold between 10 N and 50 N [48,49,50]. Therefore, the 15 N threshold set in the current study is expected to accurately detect the stance phase. The GRFs calculated for each sensor during the gait cycle were then time-normalized to 0% at heel strike and 100% at toe-off, and finally, the data were normalized by the participant’s body weight. The time-normalize GRF data for the stance phase (from 0% to 100%) were averaged into 10% intervals to facilitate comparison across the stance phase. This yielded ten data points representing the GRF at each 10% interval of the stance phase (Figure 4A,B).

### 2.6. Ground Reaction Force Variability

The GRF variability for each stance phase interval was calculated as follows: Initially, the standard deviation (SD) of the body-weight-normalized GRF was calculated across the 30 gait cycles. To quantify GRF variability within each stance sub-phase interval, the GRF SD data were divided into 10% intervals [46], and the mean SD for each sub-phase was subsequently calculated. This yielded ten data points representing the GRF at each 10% interval of the stance phase (Figure 4C,D).

### 2.7. Statistical Analysis

Statistical analyses in this study were performed using JMP Pro 17 (SAS Institute Inc., Cary, NC, USA) and MATLAB version R2023b. Before compared between groups, the distribution of the data was verified using the Shapiro–Wilk test, and the homogeneity of variances was checked using Levene’s test. An unpaired *t*-test was used to compare the following between post-stroke patients and controls: age, height, body weight, and gait speed. A χ^2^ test was used to compare sex and cane usage between the groups.

To clarify the differences in GRF and GRF variability across the stance sub-phase during walking, we performed a three-group comparison using the paretic side, the non-paretic side, and the age-matched control’s left side as factors. A one-way analysis of variance (ANOVA) was used if the normal distribution and homogeneity of variances were confirmed. Otherwise, the Kruskal–Wallis test was employed. After performing between-group comparisons, we conducted a sensitivity analysis using a linear mixed model (LMM) with walking speed as the fixed effect. This was performed because GRF and GRF variability are known to be associated with walking speed during the stance phase [51,52]. The effect size and statistical power were calculated for each value. The effect size (η^2^) was defined as follows: large (≥0.14), medium (≥0.06), and small (≥0.01) [53]. Sufficient statistical power (1 − β) was defined as ≥0.80 [53]. A Tukey–Kramer multiple comparison test was performed for the post hoc analysis.

Spearman’s rank correlation coefficient, adjusted for gait speed, was used to examine the association between GRF variability of the significant stance sub-phase and total and sub-item scores on the BBS. The significant sub-phases were those identified in the comparison among the paretic side, the non-paretic side, and the age-matched control’s left side.

The BBS sub-items on which all post-stroke participants achieved the maximum score were excluded from the correlation analysis. The correlation coefficients were classified as weak (r < 0.50), moderate (0.50 ≤ r ≤ 0.79), and strong (0.80 ≤ r) [54].

Post-stroke patients were divided into faller group and non-faller group based on their fall status. Wilcoxon rank-sum test was used to compare the physical characteristics and clinical assessment results of these two groups. Furthermore, for stance sub-phase showing significant differences compared to the control’s left side, we performed group comparisons (faller group vs. non-faller group) for GRF and GRF variability. For this comparison, the difference in characteristics was statistically adjusted by first calculating the residuals of the least squares method for any significantly different characteristics between the two groups and subsequently comparing these residuals using the Wilcoxon rank-sum test. The effect size (r) was defined as follows: large (≥0.50), medium (≥0.30), and small (≥0.10) [55].

The *p*-value < 0.05 was considered significant. Due to the small sample size, the Benjamini–Hochberg correction was applied to the between-group comparison of fallers and non-fallers in post-stroke patients, and the corrected *p*-value was used to determine the difference between the groups.

## 3. Results

### 3.1. Participant Characteristics

A total of 20 post-stroke patients and 20 age-matched controls met the inclusion criteria and participated in the measurements. A robust regression analysis based on Huber estimation was performed to identify outliers in the AP and V GRF variability. Data with residuals greater than four times the standard deviation from the mean were excluded as outliers. The final analysis included 16 post-stroke patients and 19 age-matched controls. The Four post-stroke patients who were excluded exhibited insufficient clearance on the paretic side during gait measurement led to unreliable GRF data (the AP GRF data, specifically, exhibited either a positive value in the initial sub-phases or a bifid positive peak during the late stance phase), which hindered accurate measurement. Therefore, in addition to the standard deviation criteria, these four participants were ultimately excluded after visual confirmation of the GRF data. No adverse events occurred during gait measurement. Table 1 summarizes the demographic and clinical characteristics of the stroke survivors and age-matched controls. The groups were well-matched for age, sex ratio, height, and body weight. Table 1 showed that cane use was significantly higher and gait speed was significantly lower in the stroke group. Specifically, five post-stroke patients and zero controls used a cane during measurement, and one post-stroke patient and zero controls regularly used a lower limb orthosis in daily life. Clinical diagnoses, along with FMA-L, BBS, and SIAS scores for the post-stroke patients, are also presented.

### 3.2. Comparison of GRF Across Stance Sub-Phase Among the Paretic Side, Non-Paretic Side and Control’s Left Side

Table A2 shows the results of comparing AP GRF among the three groups after adjusting for gait speed. Based on the results of the Shapiro–Wilk and Levene’s tests, a one-way analysis of variance (ANOVA) was performed on the 1–10%, 11–20%, 71–80%, 81–90%, and 91–100% stance sub-phase segments. AP GRF variability showed a significant intergroup difference during the 11–20% (*F* = 3.531, *p* = 0.037), 21–30% (χ^2^ = 6.316, *p* = 0.043), 31–40% (χ^2^ = 7.249, *p* = 0.027), 41–50% (χ^2^ = 8.111, *p* = 0.017), 51–60% (χ^2^ = 7.668, *p* = 0.022), 61–70% (χ^2^ = 6.650, *p* = 0.036), 71–80% (*F* = 5.293, *p* = 0.008), 81–90% (*F* = 6.343, *p* = 0.004), and 91–100% (*F* = 6.276, *p* = 0.004) stance sub-phases. The post hoc analysis showed the paretic side was significantly lower than the age-matched control’s left side in the 71–80% (*p* = 0.018), 81–90% (*p* = 0.021), and 91–100% (*p* = 0.012) sub-stance phases, and the non-paretic side was significantly lower than the age-matched control’s left side in the 41–50% (*p* = 0.032), 51–60% (*p* = 0.021), and 51–60% (*p* = 0.040) sub-stance phases. After adjusting for walking speed, the sensitivity analysis showed no significant intergroup difference.

Table A3 shows the results of comparing V GRF among the three groups and after adjusting for gait speed for sensitivity analysis. Based on the results of the Shapiro–Wilk and Levene’s tests, a one-way analysis of variance (ANOVA) was performed on the 11–20%, 71–80%, and 91–100% stance sub-phase segments. No significant differences were observed in V GRF among the paretic side, the non-paretic side, and the age-matched control’s left side at any point in the stance sub-phase.

### 3.3. Comparison of GRF Variability Across Stance Sub-Phase Among the Paretic Side, Non-Paretic Side, and Control’s Left Side

Table 2 show the results of comparing AP GRF variability across the stance sub-phase for the three groups and after adjusting for gait speed for sensitivity analysis. Based on the results of the Shapiro–Wilk and Levene’s tests, the Kruskal–Wallis test was performed on the across stance sub-phase segments. AP GRF variability showed a significant intergroup difference during the 91–100% stance sub-phase as the pre-swing phase (χ^2^ = 11.4037, *p* = 0.003). A significant intergroup difference remained even after adjusted for walking speed (*F* = 3.721, *p* = 0.038). The post hoc analysis showed the paretic side was significantly lower than the age-matched control’s left side in the 91–100% stance sub-phase (*p* = 0.030).

Table A4 show the results of comparing V GRF variability across the stance sub-phase among the three groups and after adjusting for gait speed. Based on the results of the Shapiro–Wilk and Levene’s tests, the Kruskal–Wallis test was performed on the across stance sub-phase segments. No significant differences were observed in the V GRF variability across the entire stance phase among the paretic side, the non-paretic side, and the age-matched control’s left side.

### 3.4. Association Between GRF Variability and Balance Ability Across Stance Sub-Phase in Post-Stroke Patients

Table 3 shows the results of the partial correlation analysis adjusted for gait speed between AP GRF variability and BBS scores. The analysis focused on the 91–100% stance sub-phase as the pre-swing phase of the paretic side because this stance sub-phase showed a significant difference compared to the control’s left side. No significant correlation was found between AP GRF variability during the 91–100% stance sub-phase on the paretic side and total or sub-item scores on the BBS in post-stroke patients.

### 3.5. Differences in GRF and GRF Variability Between the Faller Group and Non-Faller Group of Post-Stroke Patients

Table 4 shows the fall status and physical characteristics of the faller group and non-faller group of post-stroke patients. Four post-stroke participants were classified into the faller group. There were no significant differences between the two groups regarding age, height, body weight, cane usage, gait speed, diagnosis, days post-stroke, paretic side, use of an ankle–foot orthosis, or results for the FMA-L, BBS, Short FES-I, hip flexion, and knee extension tests in the SIAS.

The AP GRF variability was significantly lower in the faller group across several stance sub-phases: 41–50% (*p* = 0.045), 51–60% (*p* = 0.045), 61–70% (*p* = 0.045), and 91–100% (*p* = 0.045) stance sub-phases. After adjusting for sex and orthosis, the sensitivity analysis showed no significant intergroup difference (Table 5).

The AP GRF was significantly lower in the faller group across several stance sub-phases: 41–50% (*p* = 0.043), 51–60% (*p* = 0.045), 61–70% (*p* = 0.043), and 71–80% (*p* = 0.043) stance sub-phases. After adjusting for sex and orthosis, the sensitivity analysis showed no significant intergroup difference (Table A5).

The Wilcoxon rank-sum test was used because the Shapiro–Wilk and Levene’s tests indicated a lack of normal distribution and homogeneity of variances.

## 4. Discussion

This study examined differences in GRF variability across the stance phase between the paretic and non-paretic sides of post-stroke patients and the left side of age-matched controls as well as its relationship with balance ability. Our findings demonstrated that AP GRF variability on the paretic side was significantly lower than controls during the 91–100% stance phase. This finding contradicted our hypothesis. Furthermore, among post-stroke patients, those with a history of falls exhibited markedly lower AP GRF variability on the paretic side across the 41–70% and 91–100% sub-stance phase than non-fallers; after adjusting for sex and orthosis, the sensitivity analysis showed no significant intergroup difference. To our knowledge, this is the first study to reveal that reduced AP GRF variability during the late stance phase is a distinctive feature of post-stroke gait and that further reductions across multiple stance sub-phases are associated with a history of falls.

### 4.1. GRF Variability During Gait in Post-Stroke Patients

Contrary to our initial hypothesis of increased gait variability in post-stroke patients, our results revealed that AP GRF variability within the 91–100% stance sub-phase in post-stroke patients was significantly lower than age-matched control’s left side, with a large effect size and sufficient power. While previous research has shown that gait variability generally increases with walking speed in parameters such as V GRF and stride length [52], the significant difference in AP GRF variability between the paretic side and the age-matched control’s left side persisted even after adjusting for gait speed. This finding suggests that the control system prioritizes suppressing GRF variability during the pre-swing phase on the paretic side regardless of walking speed. This strategy of suppression appears consistent with reports that post-stroke patients prioritize mediolateral control during complex center-of-mass (COM) movements [56], potentially reflecting a mechanism to reduce movement variability and increase stability in the complex control phase [56]. The pre-swing phase is a complex control period [24,25,26], requiring simultaneous forward propulsion via concentric plantarflexor power and shock absorption by increasing contralateral ankle plantarflexion moment [57,58,59]. However, post-stroke patients demonstrate reduced ankle plantarflexion power during the pre-swing phase [60]. Therefore, due to impaired control of the paretic ankle plantarflexor muscles, post-stroke patients may suppress AP GRF variability to maintain gait. These findings may differentiate the current study from prior research by interpreting GRF variability through the lens of bilateral lower limb gait control mechanisms. The primary objective of this study, which was to elucidate the characteristics of GRF variability in stroke survivors, was achieved by demonstrating that their GRF variability was lower than that of controls.

### 4.2. Association Between GRF Variability During Gait and Balance Ability

We found no direct association between paretic side AP GRF variability and balance ability in post-stroke patients. While studies of patients with spinal cord injuries have shown that greater gait variability is associated with reduced balance ability [30], those analyses did not adjust for gait speed. In healthy individuals, V GRF variability increasing with increasing gait speed [52]. Furthermore, a positive correlation has been observed between BBS scores and gait speed in post-stroke patients [39]. This suggests that gait speed may act as a confounder for the relationship between AP GRF variability and the BBS. Additionally, the BBS is known to exhibit a ceiling effect when used with high-functioning subjects [61]. The mean BBS score of our stroke survivors was higher than previously reported fall-risk cut-off scores (49 points [39]); the BBS may not have been sensitive enough to reflect subtle differences among the stroke survivors in our study. Therefore, the lack of an association found between AP GRF variability and the BBS score can be attributed to two main possibilities: (1) the potential action of walking speed as a confounding factor and (2) the reduced sensitivity of the BBS due to its ceiling effect.

### 4.3. The Difference of GRF Variability Characteristics Between Faller and Non-Faller Groups

The AP GRF variability on the paretic side was significantly lower in the faller group during the 41–50%, 51–60%, 61–70%, and 91–100% sub-stance phases, results which were contrary to our hypothesis. This finding is contrary to previous research showing an increase in GRF variability when healthy individuals walk in unstable environments [32]. The required coefficient of friction (RCOF), which is calculated as the ratio of horizontal GRF to vertical GRF (the horizontal GRF/V GRF), serves as an index of fall risk due to slipping [62,63]. In healthy individuals, the RCOF increases during foot contact and lift-off, which consequently increases the risk of falling [62,64]. In the current study, the stance sub-phase where AP GRF variability was reduced approximated the points at which the RCOF was known to increase in a previous study [63]. Therefore, maintaining a low RCOF may require strict control of AP GRF values, which was related to the horizontal GRF. In fact, it has been reported that an increase in the RCOF during walking increases the incidence of falls by approximately 1.7 times in healthy individuals [62]. Furthermore, post-stroke patients who experience falls have also exhibited reduced gait speed variability [65], suggesting an association between decreased variability during walking and the occurrence of falls. The decreased AP GRF variability observed in the faller group therefore suggests a strict control of the AP GRF pattern during each gait cycle, thereby potentially compensating to avoid fall-related factors such as slips. This suggests an adaptive strategy for post-stroke fallers: control of the ground reaction force (GRF) is reinforced during the late stance phase, when slips are most likely to occur, to prevent falls. Control of the GRF is reinforced during the late stance phase, which is the period most susceptible to slips, in order to prevent falls. Gait variability is controlled by an adaptive balance; excessive and insufficient variability are both considered maladaptive, and a certain degree of gait variability has been shown to be essential for optimal gait control [66]. The decreased anteroposterior AP GRF variability observed in this study during the late stance phase (the propulsion period) is interpreted as an adaptive control strategy that depends on the situation and is aimed at preventing slips during high-risk falling phases. Conversely, an excessive reduction in variability may indicate a lack of flexibility in motor control. Therefore, further investigation into task-dependent variability control is warranted. Nevertheless, these significant intergroup differences disappeared following a sensitivity analysis adjusted for sex and orthosis. Gait variability is influenced by differences in body size due to sex [67]. Furthermore, the daily use of an orthosis is affected by motor function in the lower limbs [68]. Collectively, these findings indicate that in the context of investigating the relationship between GRF variability and fall occurrence in post-stroke patients, it is imperative to consider the interplay between body structure and motor function. This methodological approach distinguishes our study from previous research in this field. The objectives of this study were achieved by clarifying the characteristics of GRF variability in fallers and by elucidating the necessity of considering physical characteristics and orthotic usage in interpreting these findings.

### 4.4. GRF During Gait in Post-Stroke Patients

The AP GRF revealed intergroup differences compared to healthy controls across the 11–100% sub-phases, although the statistical power analysis (1 − β) indicated that only the 71–80%, 81–90%, and 91–100% sub-phases had sufficient power. The significant intergroup differences observed in these sub-stance phases disappeared in the sensitivity analysis when walking speed was included as a covariate. A previous study has shown that the AP GRF in the terminal stance and pre-swing phase increased with greater walking speed [69]. The walking speed of the stroke patients in this study was significantly lower than that of the control group. Therefore, the disappearance of the significant intergroup differences in AP GRF following the sensitivity analysis (adjusted for walking speed) is plausible. This outcome suggests that AP GRF is closely linked to walking speed, which is consistent with previous studies. Conversely, no significant intergroup difference was observed in V GRF. A previous study of post-stroke patients has found an association between the BBS and V GRF [70]. Given that the stroke patients in the current study had relatively high BBS scores, it is possible that a significant difference in V GRF values was not observed, even before adjusting for walking speed. Although this was not a primary hypothesis, consistent with previous studies, our results indicated that GRF during walking is associated with walking speed and balance ability.

## 5. Limitations of the Study and Future Research

This study has several limitations: (1) Our cohort of stroke survivors presented with relatively mild motor and balance impairments. Given that the severity of motor paralysis significantly influences GRF [71], the trends in GRF variability may differ in patients with more severe impairments. Future studies should investigate GRF variability stratified by the severity of motor dysfunction. Furthermore, using highly sensitive balance assessments, such as the Mini-BESTest, may facilitate novel findings regarding the relationship between GRF variability and balance ability. (2) Although a significant difference was found between the faller and non-faller groups, the faller group had a smaller sample size. However, the result of this study demonstrated a sufficient effect size. While previous studies have shown a relationship between falling and gait variability [29,30], increasing the sample size and balancing the groups in future research may reveal new insights. (3) We did not standardize the condition of cane use during measurement. Given that cane use can improve walking speed and stride in patients with speeds between 0.4 and 0.8 m/sec [72], future study designs should account for this influence by setting conditions with and without cane use. (4) The fixed sizing of the sensor shoes used may not have perfectly matched every participant’s foot dimensions. Given that the sensors are placed directly on the sole of the shoe, potential errors may exist between the anatomically defined sensor location and the actual sensor position. However, the portability of the sensor shoes allowed us to measure continuous variability over 30 gait cycles, a novel aspect of this study that is difficult to replicate using traditional force plates for measurement. (5) We used only the left-side GRF data from the healthy controls for comparison. While comparing both sides of healthy controls with the paretic and non-paretic sides of stroke patients might yield different trends, previous studies report that gait symmetry is maintained in older adults with high walking speeds [73]. Given that the walking speed of the healthy participants in this study was relatively fast (1.25 m/s), their gait likely exhibited symmetry. Therefore, using only the left side for group comparison is expected to have a minimal impact on our overall findings.

## 6. Conclusions

In post-stroke patients, AP GRF variability on the paretic side during the 91–100% stance sub-phase was significantly lower than in the controls, and no significant correlation was found between AP GRF variability in this stance sub-phase and the BBS score. Furthermore, post-stroke patients who experienced falls exhibited a significant reduction in AP GRF variability during the 41–50%, 51–60%, 61–70%, and 91–100% stance sub-phases on the paretic side. These findings suggested that post-stroke patients primarily control AP GRF variability during the critical transition from the stance phase to the swing phase. In contrast, in those who experienced falls, the suppression of AP GRF variability across approximately half of the stance phase intervals may suggest that this excessive suppression of variability reinforces gait control at points susceptible to slips.

## Figures and Tables

**Table 1:** Characteristics of post-stroke patients and age-matched controls.

	Post-Stroke Patients	Controls	Test Statistics	*p*-Value
Number	16	19		
Age (years) ^a,b^	72.19 (8.54)	68.63 (5.73)	1.467	0.151
Sex (male/female) ^c^	10/6	10/9	0.347	0.556
Height (cm) ^a,b^	157.14 (12.34)	161.33 (8.82)	−1.168	0.251
Weight (kg) ^a,b^	55.68 (8.60)	56.65 (10.57)	−0.294	0.77
Cane (T-cane/none) ^c^	5/11	0/19	8.883	0.003 *
Gait speed (m/sec) ^a,b^	0.81 (0.32)	1.25 (0.11)	−5.577	<0.001 *
Diagnosis				
Infarction ^d^	12 (75%)	-		-
Hemorrhage ^d^	4 (25%)	-		-
Paretic side				
Right ^d^	9 (56%)	-		-
Left ^d^	7 (44%)	-		-
Time post-stroke (days) ^a^	91.56 (36.13)	-		-
Fugl–Meyer Assessment ^a^	30.94 (3.00)	-		-
Berg Balance scale ^a^	51.69 (4.13)	-		-
Stroke Impairment Assessment Set (1/2/3/4/5)		-		-
Hip flexion test	0/0/1/4/11	-		-
Knee extension test	0/0/2/5/9	-		-
Ankle dorsiflexion test	0/0/0/5/11	-		-

^a^: Mean (standard deviation), ^b^: Unpaired *t*-test, ^c^: Chi-square test, ^d^: Number (percentage), *: *p* < 0.05.

**Table 2:** The comparison of anterior–posterior ground reaction force variability during gait among the paretic side, the non-paretic side in post-stroke patients, and age-matched control’s left side, adjusted for gait speed.

	Paretic Side	Non-Paretic Side	Control	Unadjusted Test Statistics	Unadjusted *p*-Value	Adjusted *F* Value	Adjusted *p*-Value	Adjusted η^2^	Adjusted 1 – β	Adjusted 95% CI
Number	16		19							
Stance phase (%)										
1–10%	−0.445 (−0.414)	−0.411 (0.387)	−0.284 (0.349)	1.435	0.488	0.971	0.389	0.042	0.227	0.020–0.075
11–20%	−1.027 (0.800)	−0.967 (0.654)	−0.487 (0.542)	0.569	0.752	1.068	0.355	0.027	0.163	0.022–0.081
21–30%	−1.222 (0.999)	−1.192 (0.776)	−0.605 (0.704)	1.458	0.482	1.111	0.341	0.029	0.169	0.023–0.083
31–40%	−1.222 (1.131)	−1.226 (0.826)	−0.543 (0.804)	1.232	0.540	1.324	0.280	0.041	0.227	0.027–0.094
41–50%	−1.128 (1.274)	−1.152 (0.836)	−0.324 (0.910)	1.249	0.536	1.798	0.181	0.062	0.336	0.036–0.118
51–60%	−1.100 (1.443)	−1.054 (0.935)	−0.148 (1.124)	1.444	0.486	1.171	0.322	0.039	0.215	0.024–0.086
61–70%	−1.049 (1.701)	−0.895 (1.142)	0.178 (1.357)	1.392	0.499	0.473	0.627	0.017	0.113	0.010–0.048
71–80%	−0.668 (1.845)	−0.611 (1.322)	0.815 (1.435)	0.084	0.959	0.240	0.789	0.001	0.054	0.005–0.035
81–90%	0.324 (1.350)	0.139 (1.043)	1.387 (0.989)	1.151	0.563	1.327	0.283	0.021	0.135	0.027–0.094
91–100%	0.479 (0.551)	0.463 (0.661)	1.167 (0.781)	11.404	0.003 *	3.721	0.038 *	0.232	0.937	0.072–0.202

Mean (Standard deviation), adjusted for group and gait speed using a linear mixed model. 95% CI: 95% confidence interval, *: *p* < 0.05.

**Table 3:** Correlation of between anterior–posterior ground rection force variability and Berg Balance scale, adjusted for gait speed.

	Partial Correlation	*p*-Value
BBS total score	0.08	0.78
Stand eye closed	0.36	0.191
Arm reaching	−0.08	0.782
Object pick up	0.23	0.403
Twist turn	−0.17	0.545
Turn 360°	0.23	0.408
Step on stool	0.28	0.316
Tandem standing	0.05	0.855
One leg standing	−0.13	0.651

Spearman’s rank correlation, BBS: Berg Balance Scale.

**Table 4:** Characteristics of faller and non-faller groups in post-stroke patients.

	Faller	Non-Faller	Test Statistics	*p*-Value
Number	4	12		
Age (years) ^a^	71.75 (11.32)	72.33 (8.03)	29	0.624
Sex (male/female) ^b^	4/0	6/6	4.534	0.0332 *
Height (cm) ^ac^	161.50 (11.32)	155.69 (12.79)	40	0.467
Weight (kg) ^ac^	55.35 (8.94)	55.79 (8.88)	32	0.856
Cane (T-cane/none) ^b^	2/2	3/9	0.834	0.361
Gait speed (m/sec) ^ac^	0.80 (0.50)	0.81 (0.26)	33	0.951
Diagnosis ^bd^			1.636	0.201
Infarction	2 (50%)	10 (83%)		
Hemorrhage	2 (50%)	2 (17%)		
Time post-stroke (days) ^ac^	94.2 (39.2)	90.7 (36.8)	35	0.870
Affected side ^b^			0.796	0.372
Right	3 (75%)	6 (50%)		
Left	1 (25%)	6 (50%)		
Orthosis (AFO/None) ^bd^	1/3	0/12	2.983	0.084
Fugl–Meyer Assessment ^ac^	29.2 (1.5)	31.5 (3.2)	24	0.287
Berg Balance scale ^ac^	52.0 (3.7)	51.6 (4.4)	32	1.000
Short Falls Efficacy Scale -International ^ac^	11.0 (4.3)	11.6 (4.6)	32	1.000
Stroke Impairment Assessment Set (1/2/3/4/5)				
Hip flexion test ^b^	0/0/0/2/2	0/0/1/2/9	2.018	0.365
Knee extension test ^b^	0/0/0/3/1	0/0/2/2/8	4.986	0.083
Ankle dorsiflexion test ^b^	0/0/0/3/1	0/0/0/2/10	4.563	0.033 *

^a^: mean (Standard deviation), ^b^: Chi-square test, ^c^: Wilcoxon rank-sum test, ^d^: Number (percentage), *: *p* < 0.05.

**Table 5:** A comparison of anterior–posterior ground reaction force variability during gait between the faller and non-faller groups in post-stroke patients, adjusted for sex and orthosis.

	Faller	Non-Faller	Unadjusted *p*	Adjusted *W*	Adjusted *p*	Adjusted Effect Size r	Adjusted 95% CI
Number	4	12					
Stance phase (%)							
1–10%	0.154 (0.047)	0.217 (0.122)	0.403	26	0.403	0.227	−0.302–0.650
11–20%	0.199 (0.024)	0.373 (0.242)	0.431	17	0.113	0.500	0.006–0.798
21–30%	0.208 (0.052)	0.413 (0.298)	0.312	18	0.120	0.470	−0.034–0.783
31–40%	0.198 (0.052)	0.428 (0.272)	0.091	19	0.132	0.440	−0.072–0.768
41–50%	0.208 (0.051)	0.431 (0.236)	0.045 *	17	0.113	0.500	0.006–0.798
51–60%	0.212 (0.053)	0.457 (0.253)	0.045 *	16	0.113	0.531	0.047–0.813
61–70%	0.187 (0.040)	0.514 (0.270)	0.045 *	16	0.113	0.531	0.047–0.813
71–80%	0.300 (0.242)	0.547 (0.186)	0.169	22	0.233	0.349	−0.178–0.720
81–90%	0.467 (0.431)	0.595 (0.271)	0.312	28	0.505	0.167	−0.359–0.612
91–100%	0.158 (0.077)	0.336 (0.122)	0.045 *	23	0.254	0.318	−0.211–0.703

Mean (standard deviation), adjusted for sex and orthosis using Wilcoxon rank-sum test. 95CI: 95% confidence interval, *: *p* < 0.05.

## Data Availability

The data, including graphs, within this paper are available from the corresponding author upon reasonable request.

## Appendix A

**Table A1:** Intraclass correlation coefficients (ICC _1,2_) for anterior–posterior (AP) and vertical (V) ground reaction force values measured by the shoe sensor system.

Stance Phase (%)	Anterior–Posterior	Vertical
10%	0.604	0.491
20%	0.596	0.544
30%	0.569	0.557
40%	0.642	0.620
50%	0.720	0.675
60%	0.727	0.689
70%	0.710	0.716
80%	0.699	0.705
90%	0.735	0.714
100%	0.752	0.872

ICC _1,2_: Intraclass correlation coefficients (1,2).

**Table A2:** A comparison of the anterior–posterior ground reaction force during gait among the paretic side, the non-paretic side in post-stroke patients, and age-matched control’s left side, adjusted for gait speed.

	Paretic Side	Non-Paretic Side	Control	Unadjusted Test Statistics	Unadjusted *p*-Value	Adjusted *F* Value	Adjusted *p*-Value	Adjusted η^2^	Adjusted 1 − β	Adjusted 95% CI
Number	16		19							
Stance phase										
1–10%	−0.445 (−0.414)	−0.411 (0.387)	−0.284 (0.349)	0.884	0.420	0.569	0.572	0.035	0.203	0.012–0.053
11–20%	−1.027 (0.800)	−0.967 (0.654)	−0.487 (0.542)	3.531	0.037 *	2.277	0.121	0.128	0.658	0.045–0.14
21–30%	−1.222 (0.999)	−1.192 (0.776)	−0.605 (0.704)	6.316	0.043 *	2.309	0.118	0.116	0.605	0.046–0.142
31–40%	−1.222 (1.131)	−1.226 (0.826)	−0.543 (0.804)	7.249	0.026 *	1.823	0.180	0.119	0.617	0.037–0.119
41–50%	−1.128 (1.274)	−1.152 (0.836)	−0.324 (0.910)	8.111	0.017 *	1.638	0.213	0.138	0.697	0.033–0.11
51–60%	−1.100 (1.443)	−1.054 (0.935)	−0.148 (1.124)	7.668	0.022 *	1.765	0.190	0.133	0.679	0.035–0.116
61–70%	−1.049 (1.701)	−0.895 (1.142)	0.178 (1.357)	6.649	0.036 *	2.564	0.095	0.142	0.715	0.051–0.153
71–80%	−0.668 (1.845)	−0.611 (1.322)	0.815 (1.435)	5.293	0.008 *	2.998	0.066	0.181	0.837	0.059–0.172
81–90%	0.324 (1.350)	0.139 (1.043)	1.387 (0.989)	6.343	0.004 *	1.143	0.332	0.209	0.900	0.023–0.084
91–100%	0.479 (0.551)	0.463 (0.661)	1.167 (0.781)	6.276	0.004 *	1.060	0.360	0.207	0.897	0.022–0.080

Mean (Standard deviation), adjusted for group and gait speed using a linear mixed model. 95%CI: 95% confidence interval, *: *p* < 0.05.

**Table A3:** A comparison of the vertical ground reaction force during gait among the paretic side, the non-paretic side in post-stroke patients, and age-matched control’s left side, adjusted for gait speed.

	Paretic Side	Non-Paretic Side	Control	Unadjusted Test Statistics	Unadjusted *p*-Value	Adjusted *F* Value	Adjusted *p*-Value	Adjusted η^2^	Adjusted 1 − β	Adjusted 95% CI
Number	16		19							
Stance phase (%)										
1–10%	0.825 (0.228)	0.759 (0.209)	0.858 (0.201)	1.7456	0.4178	0.311	0.735	0.038	0.218	0.006–0.039
11–20%	2.178 (0.928)	1.800 (0.659)	2.153 (0.504)	0.6928	0.7072	2.160	0.131	0.057	0.313	0.043–0.135
21–30%	2.977 (1.033)	2.664 (0.927)	2.957 (0.793)	0.4651	0.7925	1.750	0.191	0.024	0.151	0.035–0.115
31–40%	3.379 (0.792)	3.229 (1.048)	3.412 (0.898)	1.5863	0.4524	0.582	0.565	0.008	0.080	0.012–0.054
41–50%	3.653 (0.988)	3.518 (1.176)	3.789 (0.916)	2.7982	0.2468	0.112	0.895	0.013	0.099	0.002–0.027
51–60%	4.112 (1.289)	4.204 (1.439)	4.287 (0.959)	2.5108	0.285	0.074	0.929	0.004	0.063	0.002–0.025
61–70%	4.720 (1.288)	5.383 (1.282)	4.691 (1.208)	1.7153	0.4241	0.772	0.470	0.063	0.340	0.016–0.065
71–80%	4.941 (1.193)	6.198 (1.928)	4.832 (1.420)	1.8204	0.4024	1.640	0.208	0.144	0.722	0.033–0.110
81–90%	3.621 (0.790)	4.752 (1.955)	4.104 (1.053)	2.0716	0.3549	1.710	0.196	0.106	0.561	0.034–0.113
91–100%	1.106 (0.217)	1.332 (0.999)	1.504 (0.420)	2.7742	0.2498	2.192	0.127	0.068	0.368	0.044–0.136

Mean (Standard deviation), adjusted for group and gait speed using a linear mixed model. 95% CI: 95% confidence interval.

**Table A4:** A comparison of the vertical ground reaction force variability during gait among the paretic side, the non-paretic side in post-stroke patients, and age-matched control’s left side, adjusted for gait speed.

	Paretic Side	Non-Paretic Side	Control	Unadjusted Test Statistics	Unadjusted *p*-Value	Adjusted *F* Value	Adjusted *p*-Value	Adjusted η^2^	Adjusted 1 − β	Adjusted 95% CI
Number	16		19							
Stance phase (%)										
1–10%	0.825 (0.228)	0.759 (0.209)	0.858 (0.201)	1.746	0.4178	0.140	0.870	0.016	0.114	0.003–0.029
11–20%	2.178 (0.928)	1.800 (0.659)	2.153 (0.504)	0.693	0.7072	0.090	0.914	0.004	0.063	0.002–0.026
21–30%	2.977 (1.033)	2.664 (0.927)	2.957 (0.793)	0.465	0.7925	0.027	0.974	0.003	0.063	0.001–0.022
31–40%	3.379 (0.792)	3.229 (1.048)	3.412 (0.898)	1.586	0.4524	0.396	0.676	0.024	0.149	0.008–0.044
41–50%	3.653 (0.988)	3.518 (1.176)	3.789 (0.916)	2.798	0.2468	1.001	0.378	0.045	0.253	0.02–0.077
51–60%	4.112 (1.289)	4.204 (1.439)	4.287 (0.959)	2.511	0.285	0.753	0.479	0.036	0.205	0.015–0.064
61–70%	4.720 (1.288)	5.383 (1.282)	4.691 (1.208)	1.715	0.4241	0.453	0.640	0.030	0.175	0.009–0.047
71–80%	4.941 (1.193)	6.198 (1.928)	4.832 (1.420)	1.820	0.4024	0.593	0.560	0.042	0.233	0.012–0.055
81–90%	3.621 (0.790)	4.752 (1.955)	4.104 (1.053)	2.072	0.3549	0.597	0.556	0.036	0.204	0.012–0.055
91–100%	1.106 (0.217)	1.332 (0.999)	1.504 (0.420)	2.774	0.2498	1.341	0.275	0.055	0.302	0.027–0.095

Mean (standard deviation), adjusted for group and gait speed using a linear mixed model. 95%CI: 95% confidence interval.

**Table A5:** A comparison of the anterior–posterior ground reaction force during gait between the faller and non-faller groups in post-stroke patients, adjusted for sex and orthosis.

	Faller	Non-Faller	Unadjusted *p*	Adjusted *W*	Adjusted *p*	Adjusted Effect Size r	Adjusted 95% CI
Number	4	12					
Stance phase (%)							
1–10%	−0.723 (0.217)	−0.352 (0.429)	0.181	36	0.856	0.045	−0.461–0.529
11–20%	−1.514 (0.310)	−0.865 (0.855)	0.249	37	0.847	0.076	−0.436–0.551
21–30%	−1.913 (0.594)	−0.992 (1.017)	0.181	28	0.721	0.167	−0.359–0.612
31–40%	−2.277 (0.624)	−0.871 (1.048)	0.057	25	0.505	0.258	−0.273–0.668
41–50%	−2.445 (0.738)	−0.689 (1.107)	0.043 *	21	0.325	0.379	−0.144–0.736
51–60%	−2.542 (0.646)	−0.619 (1.310)	0.045 *	19	0.325	0.440	−0.072–0.768
61–70%	−2.764 (0.581)	−0.477(1.558)	0.043 *	20	0.325	0.409	−0.108–0.752
71–80%	−2.596 (0.547)	−0.025 (1.661)	0.043 *	21	0.325	0.379	−0.144–0.736
81–90%	−0.997 (1.078)	0.764 (1.151)	0.050	22	0.326	0.349	−0.178–0.720
91–100%	0.063 (0.648)	0.618 (0.463)	0.181	30	0.839	0.106	−0.411–0.572

Mean (standard deviation), adjusted for sex and orthosis using Wilcoxon rank-sum test. 95CI: 95% confidence interval, *: *p* < 0.05.
